# Report of a Case of Radiation-Induced New-Onset Vitiligo with Collective Review of Cases in the Literature of Radiation-Related Vitiligo

**DOI:** 10.1155/2013/345473

**Published:** 2013-08-05

**Authors:** Keechilat Pavithran, Shripad Brahmanand Pande, Makuny Dinesh

**Affiliations:** ^1^Department of Medical Oncology and Hematology, Amrita Institute of Medical Sciences, P.O. AIMS Ponekkara, Kochi, Kerala 682041, India; ^2^Department of Radiation Oncology, Amrita Institute of Medical Sciences, P.O. AIMS Ponekkara, Kochi, Kerala 682041, India

## Abstract

Radiation-induced hypopigmentation consistent vitiligo has been reported in a few case reports. We report herewith a case of vitiligo at the site of radiation delivery after a lag of several months in a patient with preexisting hypothyroidism without a previous or family history of vitiligo, and review the cases reported in the literature collectively.

## 1. Introduction

Vitiligo is a disease characterized by depigmented macules in the skin that result from a melanocyte loss. Radiation-related melanocyte loss has been described in the literature and has been incriminated for the patches of vitiligo in the radiation port. 

## 2. Case Report

A 58-year-old lady presented with a lump of approximately 2 months' duration in left breast. Examination revealed a left breast lump of about 4 cm in the greatest dimension along with an axillary lymphadenopathy that was mobile. After a discussion, patient was operated on with left modified radical mastectomy. Histopathologic examination of the tumor revealed a disease consistent with T2 and N2. She had no metastases elsewhere (M0). Out of the 22 dissected axillary nodes, 5 were positive for malignancy. On the immunohistochemistry (IHC), the tumor was triple negative (negative for estrogen receptor, progesterone receptor, and Her2/neu). Postoperatively, she received adjuvant chemotherapy with 4 cycles of Adriamycin (60 mg/m^2^), and cyclophosphamide (600 mg/m^2^), followed by 4 cycles of paclitaxel (175 mg/m^2^). For the highnodal positivity consistent with N2, she was planned to have radiotherapy to the left chest wall. She received externalbeam radiotherapy (EBRT) to a dose of 50 Gy in 25 fractions starting 4 weeks after the completion of the 24-week chemotherapy course. There was no radiation recall phenomenon. Before the commencement of radiation, she had no lesions on the chest wall on the skin elsewhere. 

Approximately 9 months after the completion of radiation (the last active therapy for the cancer), the patient was found to have a depigmentation on the left chest wall congruent with area of radiation delivery. She had no pruritus in these areas. She had no previous history or family history of vitiligo or any autoimmune diseases. She had been a case of hypothyroidism with thyroxine replacement therapy. Her TSH had been in the desired range. 

**Figure 1:**
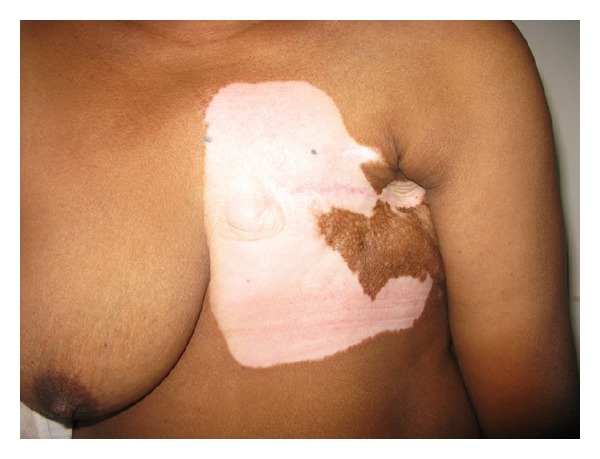
Authors' case of radiation-induced new-onset vitiligo on the chest wall in irradiated area for cancer of breast. The photograph was obtained 12 months after the onset of hypopigmentation.

The hypopigmented lesions had clear cut margins. They coalesced and persisted in all subsequent follow-up examinations. None of the lesions were raised. They were of no definite shapes. There were no telangiectasia. Biopsy or additional testing of autoantibodies could not be performed as the patient did not consent.  Figure 1 shows the area of hypopigmentation on the left chest wall; the photograph was obtained after 12 months of commencement of the depigmentation. 

At the time of scribing of this report, in the last follow-up visit some 2 months previously, the patient was well and continued to have the same hypopigmented lesions on the left chest. The lesions did not appear to have regressed or progressed to contralateral chest wall or elsewhere. The patient is on periodic follow-up for the breast cancer as well as skin lesions.

## 3. Discussion

Various hypotheses, namely, autoimmune, autotoxic, and neuronal, have been put forward to elucidate the mechanism of vitiligo. The common denominator of the condition, irrespective of the initiating mechanism, is melanocyte depletion. 

Vitiligo being induced by radiation therapy has been reported in the literature. To date, 10 cases of vitiligo at the site of radiation have been reported as on medline search. Irradiation of the skin with the resultant oxidative stress could lead to melanocyte death [1]. Vitiligo at the sites of irradiation may be linked to a proposed autocytotoxic mechanism that may occur through the inhibition of thioredoxin reductase by high extracellular calcium levels observed in the keratinocytes of vitiligo patients. High levels of thioredoxin and thioredoxin reductase have been shown to protect from the ionizing radiation-induced cell death. Thus, inhibition of thioredoxin reductase in vitiligo might account for the increased radiosensitivity of melanocytes in this disorder [2, 3]. Besides, radiation-induced apoptosis of keratinocytes leads to lower expression of the stem cell factor and basic fibroblast growth factor, among others, which possibly results in the melanocyte death [4, 5]. 

In most reports of radiation-induced vitiligo, the patients had previous history or family history of vitiligo.  Table 1 summarizes the various reports in the literature. Of 10 cases reported so far, aside from the single case of the authors, being reported herewith, seven had previous history of vitiligo, whereas one patient's past history was not available to comment upon. In these 7 patients with previous history of vitiligo, the appearance of hypopigmentation in the radiation port area was a recall of the condition they harbored. This recall could be explained on the basis of the isomorphic response or the pre-Koebner phenomenon. It is not clear whether addition of chemotherapy contributed to the development of hypopigmentation, but it was likely a risk factor. Authors' case had hypothyroidism under thyroid hormone replacement therapy. The hypothyroidism existed before the presentation to the authors, and the disease was not probed further. The disease could likely have been autoimmune, which might have had a role in the development of vitiligo in this patient, given the fact that she already had hypothyroidism. The hypothyroid disease was treated well as revealed in the preoperative thyroid function profile of the patient.

Vitiligo is a disease that has a great tendency to Koebnerize. The Boyd-Nelder classification of the Koebner phenomenon places vitiligo in category I, which is a category of diseases that truly Kobenerize; the other representatives of this category are psoriasis and lichen planus. The Boyd-Nelder category I of isomorphic response implies that the phenomenon is inseparable from pathogenesis, treatment, and prognosis of the disease.

The Koebner phenomenon can occur with a wide array of stimuli: physical (friction, trauma), chemical, biological (infective) or others [13]. 

In conclusion, in patients with past or family history of vitiligo, radiation can act as an insult, which by the Koebner phenomenon and direct damage to the melanocytes can result in development of hypopigmentation in the skin in the radiation portal, and an occasional individual can have hypopigmentation of the skin without a past history of vitiligo, as in the index case. It is worthwhile discussing with the patient with a past history of vitiligo the possible development of hypopigmentation as a rare side effect.

## Figures and Tables

**Table 1:** Summary of cases of vitiligo following radiotherapy reported in the literature.

Sr. no.	Author	Number of cases	Cancer for which radiation was given	Preexisting vitiligo	Chemotherapy	Time to hypopigmentation after completion of radiotherapy (months)	Reference
(1)	Pajonk et al.	1	Hodgkin's disease	Yes	N/A	N/A	[6]
(2)	Polat et al.	1	Nasopharyngeal carcinoma	No	Cisplatin (concurrent)	2	[7]
(3)	Levine and Ribeiro	2	Breast cancer	Yes	N/A	N/A	[8]
(4)	Koo et al.	2	Breast cancer	Yes	Yes (details N/A)	7	[9]
			Breast cancer	Yes	Yes (details N/A)	8	
(5)	Weitzen et al.	1	Breast cancer	Yes	Yes (Anthracycline based)	2	[10]
(6)	Roth	1	Melanoma	N/A	N/A	N/A	[11]
(7)	Munshi et al.	1	Breast cancer	Yes	Yes	6	[4]
(8)	Kim et al.	1	Thymoma	No	No	3	[12]
(9)	The authors	1	Breast cancer	No	Yes	9	N/A
Total cases including authors' single case		11	Breast cancer: 7 Other cancers: 4	Preexisting vitiligo: 7 None: 4	Chemo-experienced: 7 Not available: 4	Median time to develop vitiligo at radiation site: 6 months (*n* = 7)	
